# Systematic review of the complement components as potential biomarkers of pre-eclampsia: pitfalls and opportunities

**DOI:** 10.3389/fimmu.2024.1419540

**Published:** 2024-06-24

**Authors:** Andrea Balduit, Chiara Agostinis, Alessandro Mangogna, Gabriella Zito, Tamara Stampalija, Giuseppe Ricci, Roberta Bulla

**Affiliations:** ^1^ Institute for Maternal and Child Health - IRCCS “Burlo Garofolo”, Trieste, Italy; ^2^ Department of Medical, Surgical and Health Science, University of Trieste, Trieste, Italy; ^3^ Department of Life Sciences, University of Trieste, Trieste, Italy

**Keywords:** pre-eclampsia, complement system, systematic review, biomarker, pregnancy

## Abstract

The complement system (C) is a crucial component of the innate immune system. An increasing body of research has progressively shed light on the pivotal role of C in immunological tolerance at the feto-maternal interface. Excessive C activation or impaired C regulation may determine the onset of pregnancy-related pathological conditions, including pre-eclampsia (PE). Thus, several studies have investigated the presence of C components or split products in blood matrixes (*i.e.*, plasma, serum), urine, and amniotic fluid in PE. In the current study, we systematically reviewed the currently available scientific literature reporting measurements of C components as circulating biomarkers in PE, based on a literature search using Pubmed, Scopus, and Embase databases. A total of 41 out of 456 studies were selected after full-text analysis. Fourteen studies (34.1%) were identified as measuring the blood concentrations of the classical pathway, 5 (12.1%) for the lectin pathway, 28 (68.3%) for the alternative pathway, 17 (41.5%) for the terminal pathway components, and 16 (39%) for C regulators. Retrieved results consistently reported C4, C3, and factor H reduction, and increased circulating levels of C4d, Bb, factor D, C3a, C5a, and C5b-9 in PE compared to normal pregnancies, depicting an overall scenario of excessive C activation and aberrant C regulation. With evidence of C activation and dysregulation, C-targeted therapy is an intriguing perspective in PE management. Moreover, we also discussed emerging pitfalls in C analysis, mainly due to a lack of experimental uniformity and biased cohort selection among different studies and laboratories, aiming to raise a more comprehensive awareness for future standardization.

**Systematic review registration:**

https://www.crd.york.ac.uk/prospero/, identifier CRD42024503070.

## Graphical Abstract

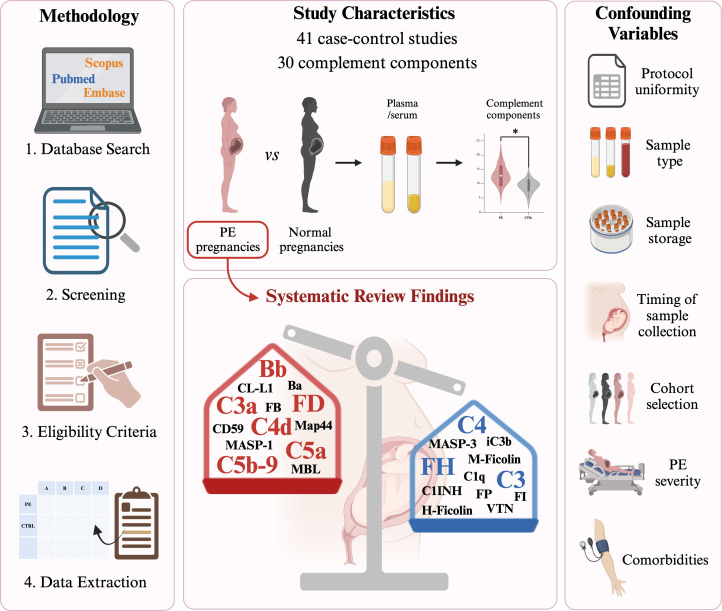

## 1 Introduction

Pre-eclampsia (PE) is a pregnancy-related disorder diagnosed by onset of hypertension (systolic blood pressure ≥ 140 mmHg and/or diastolic blood pressure ≥ 90 mmHg) after 20 weeks of gestation, associated with proteinuria (≥ 300 mg in 24 hours) and/or presence of kidney or liver dysfunction, neurological complications, hemolysis, or thrombocytopenia, and/or fetal underdevelopment. It is accounted for significant maternal and neonatal morbidity and mortality (1, 2). Based on gestational age at clinical presentation, PE is usually classified into preterm (< 37 weeks of gestation), term (≥ 37 weeks of gestation), and post-partum (from 48 hours to 6 days after delivery) (3, 4). According to onset, PE has been defined as early-onset PE (EOPE; < 34 weeks) and late-onset PE (LOPE; > 34 weeks) (5). It is now widely accepted that these two entities may differ in etiology and should be accounted for as different phenotypes of the disease (6, 7). Conversely, PE classification into mild and severe (SPE) forms has now lost significance. It can also progress to more severe complications, such as HELLP (Hemolysis, Elevated Liver enzymes, and Low Platelet count) syndrome (8) and eclampsia (9).

PE affects 5–7% of pregnant women worldwide and can occur in any pregnancy, although several associated risk factors exist. These include PE in previous pregnancies, maternal age (< 20 and ≥ 35 years), metabolic disorders, pregnancies achieved through assisted reproductive technology, and pre-existing conditions, such as chronic kidney disease, thyroid dysfunction, and systemic lupus erythematosus (SLE) (10). A family history of PE is associated with an increased risk, suggesting a genetic background (11). However, no single high-risk gene is associated with PE, reflecting the syndromic nature of the disease.

PE management involves outpatient monitoring of blood pressure and proteinuria, or potential hospitalization in more severe cases. Delivery is often the most effective intervention. Up to now, the soluble fms-like tyrosine kinase 1 (sFlt-1)/placental growth factor (PlGF) ratio is the only predictive biomarker for short-term risk of PE and adverse pregnancy outcomes (12). Additional predictive screenings via algorithms have been extensively evaluated and validated by various institutions (13). Thus, there is a critical need for serum biomarkers for early diagnosis and screening as reliable predictors of PE in the short term.

**Figure 1:**
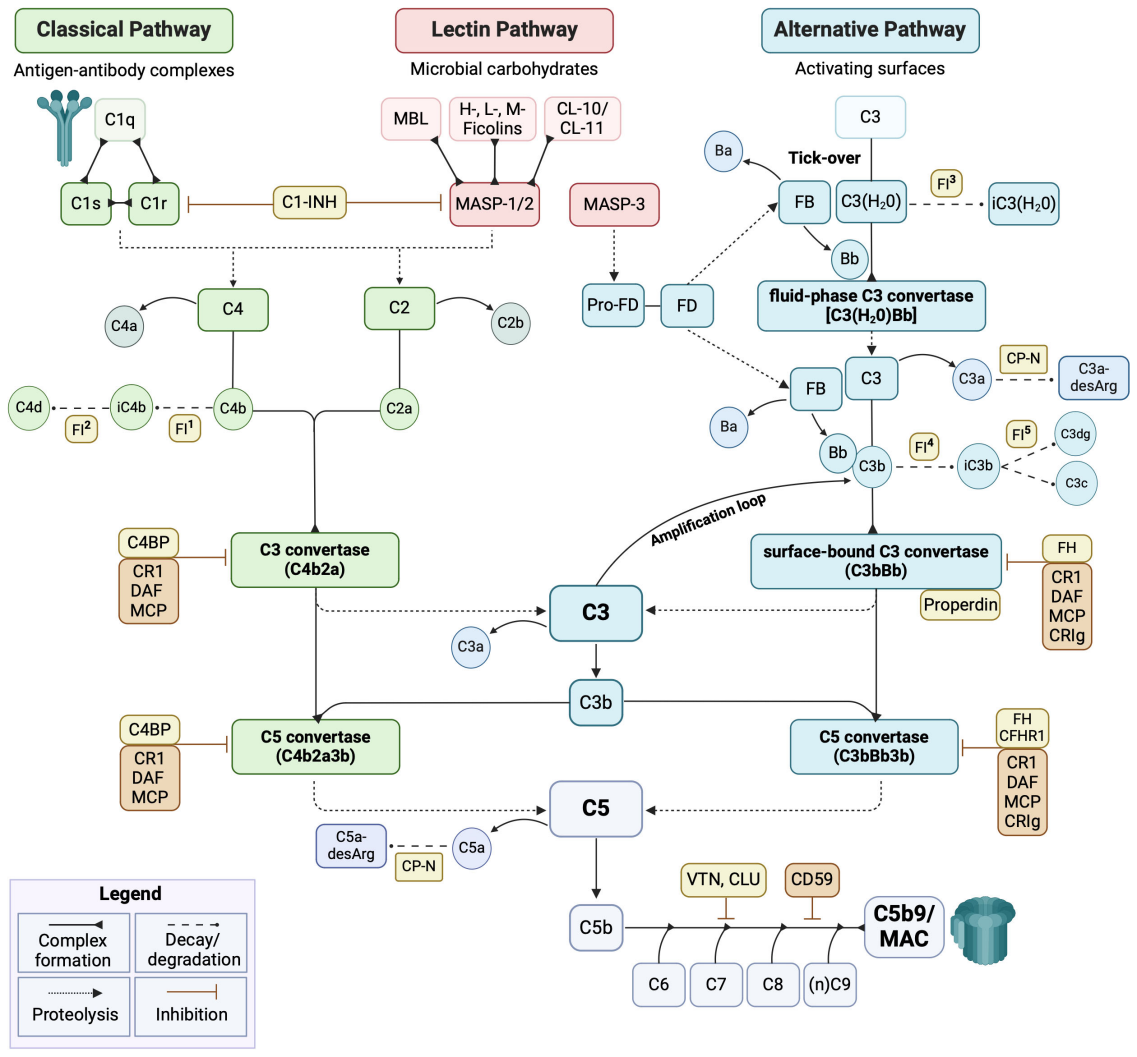
Schematic overview of the complement system cascade. The complement system (C) operates via three pathways: classical (green), lectin (pink), and alternative (cyan). The classical pathway is mainly triggered by the binding of C1q to antigen-antibody complexes. The lectin pathway is activated by mannan-binding lectin (MBL), ficolins, or collectins (CLs), which recognize microbial carbohydrates and form a complex with MASP-1 and MASP-2. The alternative pathway is constitutively active at basal levels, with C3 undergoing spontaneous hydrolysis (tick-over). All three pathways converge on the common component C3 and proceed with the terminal pathway (grey). The C activation is tightly controlled by several fluid-phase (yellow) and membrane-bound (orange) regulators. FI activity is supported by several cofactors: ^1-2)^ C4BP, CD46, CR1; ^3)^ FH/FH-L1; ^4)^ FH, FH-L1, CD46, CR1; and ^5)^ CD46, CR1. C1-INH, C1-inhibitor; C4BP, C4b-binding protein; CFHR1, complement factor H-related protein 1; CLU, clusterin; CP-N, carboxypeptidase-N; CR1, complement receptor 1; CRIg, complement receptor immunoglobulin; DAF, decay-accelerating factor; FB, factor B; FD, factor D; FH, factor H; FI, factor I; MAC, membrane attack complex; MCP, membrane cofactor protein; VTN, vitronectin. Image created with BioRender.com.

The etiology of PE is complex and multifactorial. A consistent body of evidence supports the involvement of an abnormal placentation process mainly due to immunological dysfunctions occurring at the fetal-maternal interface (13). Dysregulation of the complement system (C) is recognized as one of the main contributors to PE pathogenesis (14, 15). The C is a crucial mediator of the innate immune response, being also involved in cell homeostasis, tissue development and repair, and crosstalk with other endogenous systems (*i.e.*, renin-angiotensin, coagulation, and kinin-kallikrein systems). C consists of more than 50 fluid-phase and membrane-bound proteins. Its activation can be initiated by three distinct pathways (*i.e.*, classical, CP; lectin, LP; and alternative, AP) (**Figure 1**), converging on the common activation of the major component C3 and in the production of proinflammatory mediators, opsonization, membrane attack complex (MAC) formation, and target cell lysis (16, 17).

An accumulating body of evidence has extensively pointed out the pivotal role of C in immunological tolerance at the feto-maternal interface (18). A shift towards excessive C activation or impaired C regulation may determine the onset of pregnancy-related pathological conditions, including PE (15). An aberrant or excessive C activation in the placenta can be responsible for PE pathogenesis, leading to inflammation and endothelial dysfunction (14, 18, 19). This may be mainly due to increased circulating anaphylatoxins, enhanced deposition of C factors in the placenta, or consumption of circulating C factors, leading to placental dysfunction. C activation products can damage endothelial cells, impairing their functions and contributing to the high blood pressure and other symptoms frequently observed in PE (20). This inflammatory condition may also impact the function of other organs, such as liver and kidney, leading to complications. Interestingly, recent research has explored the possibility of targeting the C as a novel therapeutic approach for PE (21, 22).

C proteins and their activation products have been frequently found in the placenta of women with PE (23), but the relationship between circulating C components and placental C deposition remains elusive. In addition to excessive C activation leading to a simultaneous reduction in C component levels and an increase in activation products, different levels of C components between healthy pregnancies and PE may also be caused by the different hepatic synthesis due to systemic inflammation. Furthermore, one should consider the presence of a massive quantity of circulating microvesicles in the bloodstream of PE patients; they can act as decoys for C components and contribute to an aberrant concentration of C proteins and inhibitors. Thus, research interest has been focused on the evaluation of C components as PE biomarkers before the disease onset (predictive biomarkers) or during the active disease (diagnostic biomarkers). Several studies have investigated the presence of C components or split products in blood matrixes (*i.e.*, plasma, serum), urine, and amniotic fluid in PE.

This systematic review aims to evaluate the current state of the art of C components as potential circulating biomarkers in PE women, provides a general overview of C status in PE, and raises awareness about measuring C components in a reliable and standardized manner. This may help to explore novel predictive approaches and therapeutic opportunities in PE management.

## 2 Methods

### 2.1 Search methods and data sources

A systematic literature search was carried out to identify original articles reporting the values of circulating C components in PE pregnancies compared to healthy pregnancies. The search strategy was developed following the recommendations of Preferred Reporting Items for Systematic Reviews and Meta-Analyses (PRISMA) guidelines (24). PubMed, Scopus, and Embase databases were interrogated for relevant articles published until the 13^th^ March 2024. The search terms included: “preeclampsia”, “complement”, “serum”, “plasma”, “blood”, and “biomarker”. The specific search strategy is reported in the **Supplementary Material**. The review was registered in the PROSPERO database (CRD42024503070).

### 2.2 Selection of studies

Removal of duplicates and screening of records were performed using Rayyan systematic review tool (25). Two independent investigators (AB and AM) screened the articles by title and abstract, followed by full-text checking for their eligibility. Relevant articles were included if they met all the following eligibility criteria: 1) English language; 2) original peer-reviewed article; and 3) study measuring the levels of at least one C component in the blood of PE *vs* healthy women. Articles were not included if they were as follows: 1) articles published in languages other than English; 2) case reports/case series; 3) reviews, systematic reviews, meta-analyses, book chapters, conference or comment papers, prospective or retrospective studies; 4) studies reporting biomarkers in urine, cord or fetal blood; 5) studies which did not report numerical values for the measurements of circulating C components; and 6) animal studies. Disparities were resolved by a third investigator (CA).

All considered articles of interest among those identified were reported in this review.

### 2.3 Data extraction

The reported variables include: the first author’s name, year of publication, and reference of each study; the values of the biomarkers in PE and control groups, with sample size and units of measurement; *p-values* and the statistical test used; the time of sample collection and type of blood sample (*i.e.*, serum, plasma); the techniques used for biomarker measurement; notes about EOPE, LOPE, and/or SPE. The recorded statistical measures were reported as mean with standard deviation (mean ± SD) or median with interquartile range [median (IQR)].

Most articles reported a single value for a biomarker, which was included in the study. In a few research articles, multiple values obtained in different cohorts were reported for a single biomarker; in such cases, all cohorts were separately reported.

## 3 Results

### 3.1 Studies included for systematic review

**Figure 2:**
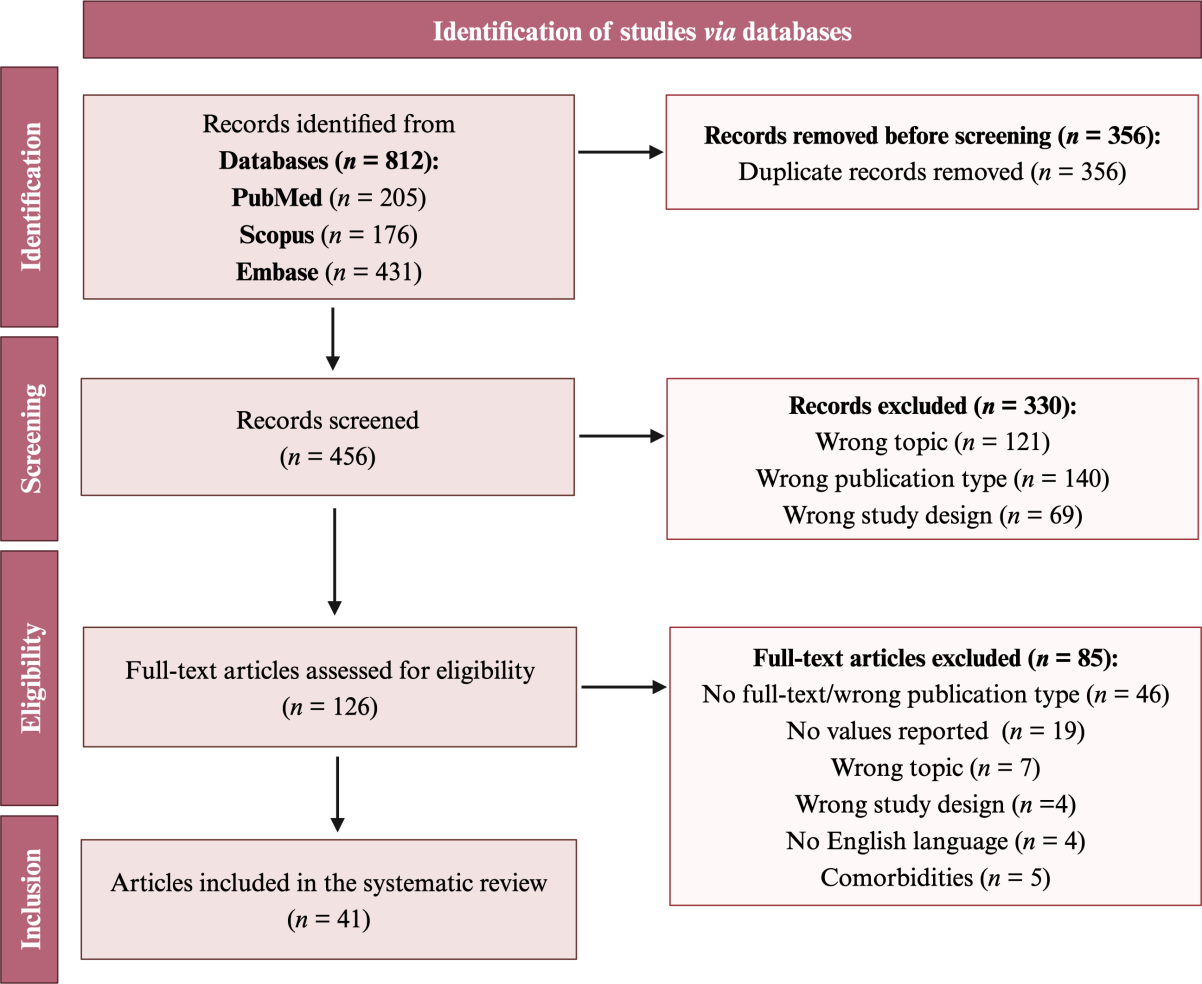
Flow diagram for article selection according to PRISMA guidelines.

The results of the literature search and selection process are illustrated in **Figure 2**. A total of 812 records were retrieved after the first search in the above-mentioned databases. Duplicates (*n* = 356) were removed via Rayyan, and the remaining 456 records were screened. During the screening phase, involving the examination of the title and abstract, 330 records were excluded for the following reasons: wrong topic (*n* = 121), wrong publication type (*n* = 140), and wrong study design (*n* = 69). Full-text screening resulted in 126 articles, which were assessed for eligibility. During the eligibility phase, 85 full-text articles were excluded since they did not meet the inclusion criteria: wrong publication type (*n* = 46), no values were reported (*n* = 19), wrong topic (*n* = 7), wrong study design (*n* = 4), languages other than English (*n* = 4), and presence of comorbidities (*n* = 5). Forty-one studies were included in this systematic review. The included papers and relative values are reported in **Tables 1**–**5**.

**Table 1:** Classical pathway.

	Factor	PE		Control		*p-value*	Test		Time	Sample	Technique	Notes
Reference		Values	n	Values	n							
Wang et al., 2021 (26)	C1q	194.0 (170.0–226.0) mg/L	49	204.0 (177.0–228.0) mg/L	147	0.346	Median (IQR)	Mann Whitney U test	upon inclusion	serum	ARCHITECT ci16200 Integrated System Chemistry/Immunology Analyzer	
Chen et al., 2021 (27)	C1q	100.9 (47.1- 449.4) µg/mL	32	92.5 (54.2- 171.9) µg/mL	48	0.001	Median (IQR)	Mann Whitney U test	n.a.	plasma	home-made ELISA	SPE^1^
He et al., 2020 (28)	C1q	192.5 ± 33.1 mg/L	21	205.0 ± 33.0 mg/L	63	0.361	Mean ± SD	t-test	~36 weeks	plasma	immunoturbidimetric assay	
Jia et al., 2019 (29)	C1q	159 (142–176) µg/mL	43	187 (148–247) µg/mL	30	<0.001	Median (IQR)	Mann Whitney U test	after admission to hospital	serum	immune transmission turbidity method on an automated AU 5800 biochemical analyzer (Beckman Coulter, Brea, CA, USA)	EOPE
Jia et al., 2019 (29)	C1q	155 (129–177) µg/mL	30	194 (179–211) µg/mL	30	<0.001	Median (IQR)	Mann Whitney U test	after admission to hospital	serum	immune transmission turbidity method on an automated AU 5800 biochemical analyzer (Beckman Coulter, Brea, CA, USA)	LOPE
He et al., 2016 (30)	C1q	88.17 (48.92–154.68) µg/mL	30	88.17 (54.19–171.87) µg/mL	30	0.337	Median (IQR)	Mann Whitney U test	n.a.	plasma	home-made ELISA	EOPE
He et al., 2016 (30)	C1q	123.34 (47.10–449.36) µg/mL	30	92.06 (42.82–136.13) µg/mL	30	0.003	Median (IQR)	Mann Whitney U test	n.a.	plasma	home-made ELISA	LOPE
Blakey et al., 2023 (31)	C4	0.20 ± 0.08 g/L	33	0.31 ± 0.08 g/L	33	<0.001	Mean ± SD	t-test	within seven days prior to delivery	serum	electrochemiluminescent immunoassays (MesoScale Diagnostics (MSD, Rockville, MD))	
He et al., 2020 (28)	C4	520.5 ± 143.9 mg/L	21	572.1 ± 173.8 mg/L	63	0.454	Mean ± SD	t-test	~36 weeks	plasma	immunoturbidimetric assay	
Jia et al., 2019 (29)	C4	139 (107–178) µg/mL	43	223 (197–269) µg/mL	30	<0.001	Median (IQR)	Mann Whitney U test	after admission to hospital	serum	immune transmission turbidity method on an automated AU 5800 biochemical analyzer (Beckman Coulter, Brea, CA, USA)	EOPE
Jia et al., 2019 (29)	C4	136 (103–180) µg/mL	30	235 (188–280) µg/mL	30	<0.001	Median (IQR)	Mann Whitney U test	after admission to hospital	serum	immune transmission turbidity method on an automated AU 5800 biochemical analyzer (Beckman Coulter, Brea, CA, USA)	LOPE
Sarween et al., 2018 (32)	C4	0.23 (0.18–0.31) g/L	88	0.27 (0.22–0.35) g/L	107	0.001	Median (IQR)	Mann Whitney U test	n.a.	serum	Hitachi Cobas 6000 Turbidimeter (Roche diagnostics, West Sussex, UK)	
Derzsy et al., 2010 (33)	C4	0.31 (0.25–0.40) g/L	60	0.28 (0.24–0.35) g/mL	60	n.s.	Median (IQR)	Mann Whitney U test	n.a.	serum	radial immunodiffusion	
Mellembakken et al., 2001 (34)	C4	0.16 (0.07–0.48) g/L	15	0.21 (0.10–0.30) g/L	19	<0.01	Median (IQR)	Mann Whitney U test	before labor	plasma	nephelometric technique	
Buyon et al., 1986 (35)	C4	28.9 ± 3.1 mg/dL	17	37 ± 1.8 mg/dL	24	<0.05	Mean ± SD	t-test	during labor	serum	radial immunodiffusion	
Massobrio et al., 1985 (36)	C4	32 ± 2.1 mg/dL	38	35 ± 1.5 mg/dL	30	n.s.	Mean ± SD	t-test	before labor	serum	radial immunodiffusion	
Hsieh and Cauchi, 1983 (37)	C4	19.1 ± 9.4 mg/dL	10	31.5 ± 5.5 mg/dL	30	<0.001	Mean ± SD	t-test	n.a.	plasma	radial immunodiffusion	
Soto et al., 2010 (38)	C4a	9124.3 (1020.3–25,940) ng/mL	54	10125.4 (850.7–32,640) ng/mL	134	n.s.	Median (IQR)	Mann Whitney U test	n.a.	plasma	ELISA (Assay Designs, Inc., Ann Arbor, MI, USA)	
Chen et al., 2021 (27)	C4d	8.43 (0.47–34.49) µg/mL	32	5.23 (0.82–11.4) µg/mL	48	0.15	Median (IQR)	Mann Whitney U test	n.a.	plasma	home-made ELISA	SPE^1^
He et al., 2016 (30)	C4d	8.62 (0.35–32.52) µg/mL	30	6.31 (1.69–11.41) µg/mL	30	1.0	Median (IQR)	Mann Whitney U test	n.a.	plasma	ELISA (Quidel)	EOPE
He et al., 2016 (30)	C4d	10.56 (0.47–34.49) µg/mL	30	4.88 (0.82–15.58) µg/mL	30	0.014	Median (IQR)	Mann Whitney U test	n.a.	plasma	ELISA (Quidel)	LOPE
Halmos et al., 2012 (39) & Derzsy et al., 2010 (33)	C4d	0.16 (0.10–0.21) µg/mL	60	0.11 (0.08–0.15) µg/mL	60	<0.05	Median (IQR)	Mann Whitney U test	n.a.	plasma	ELISA (Quidel)	

EOPE, early-onset pre-eclampsia; IQR, interquartile range; LOPE, late-onset pre-eclampsia; n.a., not available; SD, standard deviation; SPE, severe pre-eclampsia. ^1^SPE defined according to the 2013 American College of Obstetricians and Gynecologists (ACOG) hypertension in pregnancy guidelines (40).

**Table 2:** Lectin pathway.

	Factor	PE		Control		*p-value*	Test		Time	Sample	Technique	Notes
Reference		Values	n	Values	n							
Larsen et al., 2019 (41)	CL-L1	668 (602–714) ng/mL	54	551 (490–636) ng/mL	51	<0.0001	Median (IQR)	Mann Whitney U test	37 weeks (CTRL)/before delivery (PE)	serum	immunofluorimetric assay	
Larsen et al., 2019 (41)	H-ficolin	20,338 (17,793–22,134) ng/mL	54	28,412 (23,750–32,675) ng/mL	51	<0.0001	Median (IQR)	Mann Whitney U test	37 weeks (CTRL)/before delivery (PE)	serum	immunofluorimetric assay	
Larsen et al., 2019 (41)	M-ficolin	3,804 (3,179–4,978) ng/mL	54	4,780 (3,692–5,891) ng/mL	51	0.005	Median (IQR)	Mann Whitney U test	37 weeks (CTRL)/before delivery (PE)	serum	immunofluorimetric assay	
Larsen et al., 2019 (41)	MAp19	635 (523–679) ng/mL	54	626 (514–730) ng/mL	51	0.99	Median (IQR)	Mann Whitney U test	37 weeks (CTRL)/before delivery (PE)	serum	immunofluorimetric assay	
Larsen et al., 2019 (41)	MAp44	3,116 (2,704–3,462) ng/mL	54	2,707 (2,424–3,062) ng/mL	51	0.005	Median (IQR)	Mann Whitney U test	37 weeks (CTRL)/before delivery (PE)	serum	immunofluorimetric assay	
Larsen et al., 2019 (41)	MASP-1	36,057 (29,831–43,815) ng/mL	54	24,123 (17,124–29,973) ng/mL	51	<0.0001	Median (IQR)	Mann Whitney U test	37 weeks (CTRL)/before delivery (PE)	serum	immunofluorimetric assay	
Larsen et al., 2019 (41)	MASP-2	515 (391–642) ng/mL	54	556 (448–716) ng/mL	51	0.40	Median (IQR)	Mann Whitney U test	37 weeks (CTRL)/before delivery (PE)	serum	immunofluorimetric assay	
Larsen et al., 2019 (41)	MASP-3	4,848 (4,110–5,765) ng/mL	54	5,515 (4,481–6,252) ng/mL	51	0.03	Median (IQR)	Mann Whitney U test	37 weeks (CTRL)/before delivery (PE)	serum	immunofluorimetric assay	
Chen et al., 2021 (27)	MBL	3058 (130.4–23,384) ng/mL	32	3293 (654.4- 5182) ng/mL	48	0.72	Median (IQR)	Mann Whitney U test	n.a.	plasma	home-made ELISA	SPE^1^
He et al., 2020 (28)	MBL	4965.2 (3580.6−6530.4) ng/mL	21	4278.0 (1508.9−7464.8) ng/mL	63	0.6	Median (IQR)	Mann Whitney U test	~36 weeks	plasma	home-made ELISA	
Larsen et al., 2019 (41)	MBL	2094 (791–3588) ng/mL	54	1825 (701–3250) ng/mL	51	0.84	Median (IQR)	Mann Whitney U test	37 weeks (CTRL)/before delivery (PE)	serum	immunofluorimetric assay	
He et al., 2016 (30)	MBL	3000.20 (83.82–23 383.84) ng/mL	30	3361.18 (654.37–4173.32) ng/mL	30	1.100	Median (IQR)	Mann Whitney U test	n.a.	plasma	home-made ELISA	EOPE
He et al., 2016 (30)	MBL	3176.56 (111.98–14 452.08) ng/mL	30	3039.76 (26.07–5182.46) ng/mL	30	1.210	Median (IQR)	Mann Whitney U test	n.a.	plasma	home-made ELISA	LOPE
Than et al., 2008 (42)	MBL	1128 (13.6–11870.0) ng/mL	99	886.3 (6.1–7805.1) ng/mL	187	<0.01	Median (IQR)	Mann Whitney U test	n.a.	plasma	ELISA (Antibody-Shop A/ S, Gentofte, Denmark).	

EOPE, early-onset pre-eclampsia; IQR; interquartile range; LOPE, late-onset pre-eclampsia; n.a., not available; SD, standard deviation; SPE, severe pre-eclampsia. ^1^SPE defined according to the 2013 American College of Obstetricians and Gynecologists (ACOG) hypertension in pregnancy guidelines (40).

**Table 3:** Alternative pathway.

	Factor	PE		Control		p-value	Test		Time	Sample	Technique	Notes
Reference		Values	n	Values	n							
Blakey et al., 2023 (31)	Ba	150 (119–223) ng/mL	33	113 (89–148) ng/mL	33	0.012	Median (IQR)	Mann Whitney U test	within seven days prior to delivery	plasma	electrochemiluminescent immunoassays (MesoScale Diagnostics (MSD, Rockville, MD))	
Blakey et al., 2023 (31)	Ba	165 (117–268) ng/mL	35	151 (113–198) ng/mL	35	0.310	Median (IQR)	Mann Whitney U test	within seven days prior to delivery	plasma	electrochemiluminescent immunoassays (MesoScale Diagnostics (MSD, Rockville, MD))	
Chen et al., 2021 (27)	Bb	0.62 (0.19–3.32) µg/mL	32	0.44 (0.24–0.82) µg/mL	48	<0.01	Median (IQR)	Mann Whitney U test	n.a.	plasma	home-made ELISA	SPE^1^
Jia et al., 2019 (29)	Bb	491 (423–669) ng/mL	43	389 (312–470) ng/mL	30	0.001	Median (IQR)	Mann Whitney U test	after admission to hospital	serum	immune transmission turbidity method on an auto- mated AU 5800 biochemical analyzer (Beckman Coulter, Brea, CA, USA)	EOPE
Jia et al., 2019 (29)	Bb	503 (367–773) µg/mL	30	374 (323–418) µg/mL	30	0.003	Median (IQR)	Mann Whitney U test	after admission to hospital	serum	immune transmission turbidity method on an auto- mated AU 5800 biochemical analyzer (Beckman Coulter, Brea, CA, USA)	LOPE
He et al., 2016 (30)	Bb	0.62 (0.21–3.55) µg/mL	30	0.45 (0.24–0.59) µg/mL	30	<0.001	Median (IQR)	Mann Whitney U test	n.a.	plasma	ELISA (Quidel)	EOPE
He et al., 2016 (30)	Bb	0.68 (0.19–4.10) µg/mL	30	0.43 (0.26–0.82) µg/mL	30	0.027	Median (IQR)	Mann Whitney U test	n.a.	plasma	ELISA (Quidel)	LOPE
Velickovic et al., 2015 (43)	Bb	1.26 ± 0.60 µg/ml	38	0.98 ± 0.45 µg/ml	253	0.003	Mean ± SD	t-test	upon admission to hospital for delivery	plasma	ELISA (Quidel)	
Hoffman et al., 2013 (44)	Bb	1.45 ± 1.03 µg/ml	24	0.65 ± 0.23 µg/ml	20	<0.001	Mean ± SD	t-test	prior to delivery	plasma	ELISA (Quidel)	
Derzsy et al., 2010 (33)	Bb	0.12 (0.10–0.14) µg/mL	60	0.11 (0.09–0.15) µg/mL	60	n.s.	Median (IQR)	Mann Whitney U test	n.a.	plasma	ELISA (Quidel)	
Lynch et al., 2008 (45)	Bb	0.8 ± 0.3	32	0.68 ± 0.2	669	0.02	Mean ± SD	t-test	before 20 weeks’ gestation	plasma	home-made ELISA	
Blakey et al., 2023 (31)	C3	1.90 ± 0.39 g/L	33	2.36 ± 0.39 g/L	33	<0.001	Mean ± SD	t-test	within seven days prior to delivery	plasma	electrochemiluminescent immunoassays (MesoScale Diagnostics (MSD, Rockville, MD))	
Jia et al., 2019 (29)	C3	1120 (1019–1311) µg/mL	43	1438 (1264–1603) µg/mL	30	<0.001	Median (IQR)	Mann Whitney U test	after admission to hospital	serum	immune transmission turbidity method on an automated AU 5800 biochemical analyzer (Beckman Coulter, Brea, CA, USA)	EOPE
Jia et al., 2019 (29)	C3	1196 (1012–1340) µg/mL	30	1434 (1271–1602) µg/mL	30	<0.001	Median (IQR)	Mann Whitney U test	after admission to hospital	serum	immune transmission turbidity method on an automated AU 5800 biochemical analyzer (Beckman Coulter, Brea, CA, USA)	LOPE
Sarween et al., 2018 (32)	C3	1.73 ± 0.34 g/L	88	1.74 ± 0.31 g/L	107	0.838	Mean ± SD	t-test	n.a.	serum	Hitachi Cobas 6000 Turbidimeter (Roche diagnostics, West Sussex, UK)	
Derzsy et al., 2010 (33)	C3	1.56 (1.45–1.78) g/L	60	1.79 (1.60–1.91) g/L	60	<0.05	Median (IQR)	Mann Whitney U test	n.a.	serum	radial immunodiffusion	
Ari et al., 2009 (46)	C3	1.45 ± 0.26 g/L	21	1.46 ± 0.18 g/L	24	n.s.	Mean ± SD	One-way ANOVA	n.a.	serum	automated Dade Behring Nephelometer II (Dade Behring Marburg GmbH, Marburg, Germany)	
Mellembakken et al., 2001 (34)	C3	1.31 (1.01–1.60) g/L	15	1.20 (1.03–1.36) g/L	19	n.s.	Median (IQR)	Mann Whitney U test	before labor	plasma	nephelometric technique	
Buyon et al., 1986 (35)	C3	162 ± 4 mg/dL	17	165 ± 4 mg/dL	24	n.s.	Mean ± SD	t-test	during labor	serum	radial immunodiffusion	
Massobrio et al., 1985 (36)	C3	118 ± 4.3 mg/dL	38	116 ± 4mg/dL	30	n.s.	Mean ± SD	t-test	before labor	serum	radial immunodiffusion	
Chen et al., 2021 (27)	C3a	1889 (148.5–23,335) ng/mL	32	34.6 (9.86–308.1) ng/mL	48	<0.01	Median (IQR)	Mann Whitney U test	n.a.	plasma	in-house ELISA	SPE^1^
He et al., 2020 (28)	C3a	257.7 (214.7−398.1) ng/mL	21	383.1 (321.3−448.2) ng/mL	63	0.16	Median (IQR)	Mann Whitney U test	~36 weeks	plasma	ELISA (Quidel Corporation, San Diego, CA, USA)	
Wiles et al., 2018 (47)	C3a	43.7 (29.6–60.2) ng/mL	18	34.2 (30.2–50.3) ng/mL	20	0.48	Median (IQR)	Mann Whitney U test	at routine outpatient attendance	plasma	ELISA (Hycult Biotech HK354)	
Ma et al., 2018 (48)	C3a	30.1 ± 3.3 µg/mL	24	25. 9 ± 2.1 µg/mL	32	0.28	Mean ± SEM	t-test	n.a.	serum	ELISA (BD Biosciences)	
He et al., 2016 (30, 49)	C3a	679.73 (14.32–26,821.68) ng/mL	30	38.63 (14.43–308.15) ng/mL	30	<0.001	Median (IQR)	Mann Whitney U test	n.a.	plasma	ELISA (Quidel Corporation, San Diego, CA, USA),	EOPE
He et al., 2016 (30, 49)	C3a	4,018.80 (67.64–26,515.6) ng/mL	30	34.80 (9.86–162.26) ng/mL	30	<0.001	Median (IQR)	Mann Whitney U test	n.a.	plasma	ELISA (Quidel Corporation, San Diego, CA, USA),	LOPE
Ye et al., 2015 (50)	C3a	701.39 ± 263.55 ng/mL	52	29.8 ± 5.3 ng/mL	60	<0.05	Mean ± SD	t-test	prior to delivery	serum	ELISA (USCN Life Science Inc., Wuhan, China)	SPE^1^
Burwick et al., 2013 (51)	C3a	3373 ± 916 ng/mL	25	3219 ± 548 ng/mL	25	n.s.	Mean ± SD	t-test	day of enrollment	plasma	ELISA (BD Biosciences)	SPE^2^
Denny et al., 2013 (52)	C3a	63.8 ± 4.37 ng/mL	72	54.9 ± 1.62	43	n.s.	Mean ± SD	t-test	prior to delivery	plasma	ELISA (USCN Life Science Inc., Wuhan, China)	
Halmos et al., 2012 (39) & Derzsy et al., 2010 (33)	C3a	1358 (854.8–2142) ng/mL	60	751.6 (194.6–1660) ng/mL	60	<0.05	Median (IQR)	Mann Whitney U test	n.a.	plasma	ELISA (Quidel)	
Soto et al., 2010 (38)	C3a	2127.4 (698.6–15,820) ng/mL	54	2364.7 (557.9–6642.7) ng/mL	134	n.s.	Median (IQR)	Mann Whitney U test	n.a.	plasma	ELISA (Assay Designs, Inc., Ann Arbor, MI, USA)	
He et al., 2020 (28)	C3c	1503.0 ± 332.1 mg/L	21	1653 ± 343.4 mg/L	63	0.288	Mean ± SD	t-test	~36 weeks	plasma	immunoturbidimetric assay	
Griffin, 1983 (53)	C3c	72 ± 33.5 mg/dL	15	63.8 ± 17.2 mg/dL	18	n.s.	Mean ± SD	t-test	before labor	plasma	radial immunodiffusion	
Massobrio et al., 1985 (36)	C3d	6 ± 0.6 mg/dL	38	4 ± 0.8 mg/dL	30	n.s.	Mean ± SD	t-test	before labor	plasma	radial immunodiffusion	
Wang et al., 2021 (26)	Factor B	353.5 (328.5–369.3) mg/L	49	333.0 (308.0–365.0) mg/L	147	0.036	Median (IQR)	Mann Whitney U test	upon inclusion	serum	fully automated ARCHITECT ci16200 Integrated System Chemistry/Immunology Analyzer	
He et al., 2020 (28)	Factor B	548.2 ± 83.8 mg/L	21	595.0 ± 118.8 mg/L	63	0.311	Mean ± SD	t-test	~36 weeks	plasma	immunoturbidimetric assay	
Assaf et al., 2024 (54)	Factor D	34.32 ± 7.28 ng/mL	30	15.93 ± 2.36 ng/mL	29	<0.001	Mean ± SD	t-test	upon inclusion	serum	ELISA (#ELK1128; ELK Biotechnology, Co., Ltd., Wuhan)	
Assaf et al., 2024 (54)	Factor D	44.75 ± 2.81 ng/mL	30	15.93 ± 2.36 ng/mL	29	<0.001	Mean ± SD	t-test	upon inclusion	serum	ELISA (#ELK1128; ELK Biotechnology, Co., Ltd., Wuhan)	SPE^3^
David et al., 2021 (55)	Factor D	11,972 pg/mL (3240)	19	3607 pg/mL (5947)	19	<0.05	Median (IQR)	Mann Whitney U test	n.a.	serum	MILLIPLEX MAP™ Human Complement Panel	
Liu et al., 2021 (56)	Factor D	3161 ± 214.7 ng/mL	33	2153 ± 201.4 ng/mL	32	<0.01	Mean ± SD	t-test	before delivery	plasma	ELISA (RayBiotec Inc, Norcross, GA)	
Poveda et al., 2015 (57)	Factor D	5932.1 ± 2036.2 ng/mL	18	4314.5 ± 665 ng/mL	54	<0.01	Mean ± SD	t-test	~34 weeks	serum	ELISA (Abcam, USA)	
Blakey et al., 2023 (31)	iC3b	489 ± 153 ng/mL	33	606 ± 157 ng/mL	33	0.003	Mean ± SD	t-test	within seven days prior to delivery	plasma	electrochemiluminescent immunoassays [MesoScale Diagnostics (MSD, Rockville, MD)]	

EOPE, early-onset pre-eclampsia; IQR, interquartile range; LOPE, late-onset pre-eclampsia; n.a., not available; SD, standard deviation; SEM, standard error mean; SPE, severe pre-eclampsia. ^1^SPE defined according to the 2013 American College of Obstetricians and Gynecologists (ACOG) hypertension in pregnancy guidelines (40). ^2^SPE defined according to the 2002 ACOG hypertension in pregnancy guidelines (58). ^3^SPE defined according to the 2019 ACOG hypertension in pregnancy guidelines (59).

**Table 4:** Terminal pathway.

	Factor	PE		Control		*p-value*	Test		Time	Sample	Technique	Notes
Reference		Values	n	Values	n							
Chen et al., 2021 (27)	C5a	44.41 (2.91–7935.5) ng/mL	32	13.5 (2.08–278.7) ng/mL	48	<0.01	Median (IQR)	Mann Whitney U test	n.a.	plasma	home-made ELISA	SPE^1^
He et al., 2020 (28)	C5a	21.9 ± 6.8 ng/mL	21	28.9 ± 7.5 ng/mL	63	0.025	Mean ± SD	t-test	~36 weeks	plasma	ELISA (Quidel Corporation, San Diego, CA, USA)	
Burwick et al., 2019 (60)	C5a	41.0 (26.9–51.0) ng/mL	16	34.2 (28.9–38.6) ng/mL	16	n.s.	Median (IQR)	Mann Whitney U test	at delivery	plasma	ELISA (BD Biosciences, San Jose, CA)	
Wiles et al., 2018 (47)	C5a	1.25 (1.25–2.17) ng/mL	18	1.45 (1.25–3.37) ng/mL	20	0.19	Median (IQR)	Mann Whitney U test	at routine outpatient attendance	plasma	ELISA (Hycult Biotech HK349)	
Ma et al., 2018 (48)	C5a	100.4 ± 5.9 ng/mL	24	76.4 ± 3.0 ng/mL	32	0.001	Mean ± SEM	t-test	n.a.	serum	ELISA (BD Biosciences)	
He et al., 2016 (30, 49)	C5a	35.16 (1.76–7935.46) ng/mL	30	12.87 (2.31–31.70) ng/mL	30	0.003	Median (IQR)	Mann Whitney U test	n.a.	plasma	ELISA (Quidel Corporation, San Diego, CA, USA),	EOPE
He et al., 2016 (30, 49)	C5a	84.82 (3.18–3845.73) ng/mL	30	14.33 (1.91–933.20) ng/mL	30	0.001	Median (IQR)	Mann Whitney U test	n.a.	plasma	ELISA (Quidel Corporation, San Diego, CA, USA),	LOPE
Ye et al., 2015 (50)	C5a	10.37 ± 2.73 ng/mL	52	8.54 ± 2.74 ng/mL	60	<0.05	Mean ± SD	t-test	prior to delivery	serum	ELISA (USCN Life Science Inc., Wuhan, China)	SPE^1^
Burwick et al., 2013 (51)	C5a	8.2 ± 1.30 ng/mL	25	4.5 ± 0.50 ng/mL	25	<0.01	Mean ± SD	t-test	day of enrollment	plasma	ELISA (BD Biosciences)	SPE^2^
Denny et al., 2013 (52)	C5a	63.8 ± 4.37 ng/mL	72	54.9 ± 1.62	43	n.s.	Mean ± SD	t-test	prior to delivery	plasma	ELISA (USCN Life Science Inc., Wuhan, China)	
Soto et al., 2010 (38)	C5a	19.7 (4.3–94.1) ng/mL	54	12.4 (1.2–87.1) ng/mL	134	<0.05	Median (IQR)	Mann Whitney U test	n.a.	plasma	ELISA (American Laboratory Products Company, Windham, NH)	
Haeger et al., 1993 (61)	C5a	5.0 ± 0.8 ng/mL	8	4.8 ± 0.8 ng/mL	8	n.s.	Mean ± SD	t-test	time of delivery	plasma	radioimmunoassay	
Assaf et al., 2024 (54)	C5b-9	11.41 ± 1.93 ng/mL	30	7.88 ± 1.11 ng/mL	29	0.01	Mean ± SD	t-test	upon inclusion	serum	ELISA (#ELK3025; ELK Biotechnology, Co., Ltd., Wuhan)	
Assaf et al., 2024 (54)	C5b-9	22.59 ± 2.22 ng/mL	30	7.88 ± 1.11 ng/mL	29	<0.001	Mean ± SD	t-test	upon inclusion	serum	ELISA (#ELK3025; ELK Biotechnology, Co., Ltd., Wuhan)	SPE^3^
Blakey et al., 2023 (31)	C5b-9	237 (198–335) ng/mL	33	237 (185–334) ng/mL	33	0.753	Median (IQR)	Mann Whitney U test	within seven days prior to delivery	plasma	electrochemiluminescent immunoassays (MesoScale Diagnostics (MSD, Rockville, MD))	
Chen et al., 2021 (27)	C5b-9	290.2 (112.3- 616.2) ng/mL	32	110.4 (8.59–323.5) ng/mL	48	<0.01	Median (IQR)	Mann Whitney U test	n.a.	plasma	home-made ELISA	SPE^1^
He et al., 2020 (28)	C5b-9	463.4 (419.0−542.6) ng/mL	21	427.7 (302.3−632.0) ng/mL	63	0.6	Median (IQR)	t-test	~36 weeks	plasma	ELISA (Quidel Corporation, San Diego, CA, USA)	
Burwick et al., 2019 (60)	C5b-9	187 (134–234) ng/mL	16	131 (95–158) ng/mL	16	0.03	Median (IQR)	Mann Whitney U test	at delivery	plasma	ELISA (BD Biosciences, San Jose, CA)	
Wiles et al., 2018 (47)	C5b-9	294 (225–429) ng/mL	18	238 (193–360) ng/mL	20	0.30	Median (IQR)	Mann Whitney U test	at routine outpatient attendance	plasma	ELISA (Hycult Biotech HK342)	
Burwick et al., 2018 (62)	C5b-9	2900 (1396–4558) ng/mL	57	1374 (1064–2332) ng/mL	54	<0.001	Median (IQR)	Mann Whitney U test	day of enrollment	plasma	ELISA	PE
Burwick et al., 2018 (62)	C5b-9	2778 (1633–4230) ng/mL	104	1374 (1064–2332) ng/mL	54	<0.001	Median (IQR)	Mann Whitney U test	day of enrollment	plasma	ELISA	SPE^1^
Burwick et al., 2018 (62)	C5b-9	2906 (1740–5848) ng/mL	15	1378 (1096–2440) ng/mL	19	n.s.	Median (IQR)	Mann Whitney U test	day of enrollment	plasma	ELISA	EOPE
Burwick et al., 2018 (62)	C5b-9	2966 (1578–4256) ng/mL	53	1378 (1096–2440) ng/mL	19	0.003	Median (IQR)	Mann Whitney U test	day of enrollment	plasma	ELISA	EOSPE
He et al., 2016 (30, 49)	C5b-9	328.71 (112.27–1871.26) ng/mL	30	116.54 (22.00–323.54) ng/mL	30	<0.001	Median (IQR)	Mann Whitney U test	n.a.	plasma	ELISA (Quidel Corporation, San Diego, CA, USA),	EOPE
He et al., 2016 (30, 49)	C5b-9	306.56 (105.17–554.33) ng/mL	30	139.71 (8.59–317.55) ng/mL	30	<0.001	Median (IQR)	Mann Whitney U test	n.a.	plasma	ELISA (Quidel Corporation, San Diego, CA, USA),	LOPE
Burwick et al., 2013 (51)	C5b-9	444 ± 171 ng/mL	25	348 ± 172 ng/mL	25	0.04	Mean ± SD	t-test	day of enrollment	plasma	ELISA (BD Biosciences)	SPE^2^
Halmos et al., 2012 (39) & Derzsy et al., 2010 (33)	C5b-9	75.9 (50.8–116.3) ng/mL	60	59.9 (42.1–86.6) ng/mL	60	<0.05	Median (IQR)	Mann Whitney U test	n.a.	plasma	ELISA (Quidel)	
Haeger et al., 1993 (61)	C5b-9	5.0 ± 1.6 AU/mL	8	1.4 ± 0.2 AU/mL	8	<0.05	Mean ± SD	t-test	time of delivery	plasma	ELISA	

EOPE, early-onset pre-eclampsia; EOSPE, early-onset sever pre-eclampsia; IQR, interquartile range; LOPE, late-onset pre-eclampsia; n.a., not available; SD, standard deviation; SEM, standard error mean; SPE, severe pre-eclampsia. ^1^SPE defined according to the 2013 American College of Obstetricians and Gynecologists (ACOG) hypertension in pregnancy guidelines (40). ^2^SPE defined according to the 2002 ACOG hypertension in pregnancy guidelines (58). ^3^SPE defined according to the 2019 ACOG hypertension in pregnancy guidelines (59).

**Table 5:** Complement regulators.

	Factor	PE		Control		*p-value*	Test		Time	Sample	Technique	Notes
Reference		Values	n	Values	n							
Godtfredsen et al., 2022 (63)	C1-inhibitor	0.17 (0.15–0.19) g/L	117	0.17 (0.16–0.19) g/L	117	0.14	Median (IQR)	Wilcoxon matched-pairs signed rank test	upon inclusion	plasma	BN II analyser (Siemens Healthcare Diagnostics, Marburg, Germany)	
Derzsy et al., 2010 (33)	C1-inhibitor	0.19 (0.17–0.22) g/L	60	0.18 (0.16–0.20) g/L	60	n.s.	Median (IQR)	Mann Whitney U test	n.a.	serum	radial immunodiffusion	
Mellembakken et al., 2001 (34)	C1-inhibitor	0.23 (0.15- 0.55) g/L	15	0.21 (0.17–0.24) g/L	19	n.s.	Median (IQR)	Mann Whitney U test	before labor	plasma	single radial immunodiffusion (NOR-Partigen; Behringwerke A/G, Marburg, Germany)	
Hsieh and Cauchi, 1983 (37)	C1-inhibitor	11.4 ± 2.3 mg/dL	15	13.1 ± 1.5 mg/dL	32	<0.001	Mean ± SD	t-test	n.a.	serum	radial immunodiffusion	
Derzsy et al., 2010 (33)	C4b-binding protein	790.1 (614.5–980.8) µg/mL	60	823.9 (610.2–1004) µg/mL	60	n.s.	Median (IQR)	Mann Whitney U test	n.a.	serum	home-made ELISA	
Velásquez et al., 2020 (64)	CD59	32.0 (27–39) ng/mL	104	23.7 (20–31) ng/mL	54	<0.001	Median (IQR)	Mann Whitney U test	day of enrollment	plasma	ELISA (MyBioSource, San Diego, CA)	SPE^1^
Feinberg et al., 2005 (65)	CR1 /CD35	42.1 ± 4.0 ng/mL	29	37.7 ± 2.9 ng/mL	29	n.s.	Mean ± SD	t-test	just prior to labor	plasma	ELISA (T Cell Sciences, Cambridge, MA)	
Yasmin et al., 2024 (66)	Factor H	324.21 ± 131.04 μg/mL	26	556.95 ± 191.52 μg/mL	31	<0.0001	Mean ± SD	t-test	third trimester	serum	ELISA (#DY4779; R&D Systems, Inc., Minneapolis, Canada)	
Assaf et al., 2024 (54)	Factor H	33.93 ± 2.33 ng/mL	30	27.6 ± 2.41 ng/mL	29	0.02	Mean ± SD	t-test	upon inclusion	serum	ELISA (#ELK1720; ELK Biotechnology, Co., Ltd., Wuhan)	
Assaf et al., 2024 (54)	Factor H	59.02 ± 11.87 ng/mL	30	27.6 ± 2.41 ng/mL	29	<0.001	Mean ± SD	t-test	upon inclusion	serum	ELISA (#ELK1720; ELK Biotechnology, Co., Ltd., Wuhan)	SPE^3^
Wang et al., 2021 (26)	Factor H	397.5 (358.0–436.8) mg/L	49	404.0 (372.0–429.0) mg/L	147	0.664	Median (IQR)	Mann Whitney U test	upon inclusion	serum	fully automated ARCHITECT ci16200 Integrated System Chemistry/Immunology Analyzer	
He et al., 2020 (28)	Factor H	473.4 ± 70.2 mg/L	21	465.2 ± 50.8 mg/L	63	0.72	Mean ± SD	t-test	~36 weeks	plasma	immunoturbidimetric assay	
Jia et al., 2019 (29)	Factor H	351 (319–374) µg/mL	43	370 (339–398) µg/mL	30	0.009	Median (IQR)	Mann Whitney U test	after admission to hospital	serum	immune transmission turbidity method on an automated AU 5800 biochemical analyzer (Beckman Coulter, Brea, CA, USA)	EOPE
Jia et al., 2019 (29)	Factor H	354 (321–372) µg/mL	30	366 (348–408) µg/mL	30	0.031	Median (IQR)	Mann Whitney U test	after admission to hospital	serum	immune transmission turbidity method on an automated AU 5800 biochemical analyzer (Beckman Coulter, Brea, CA, USA)	LOPE
Wiles et al., 2018 (47)	Factor H	672 (567–805) µg/mL	18	1116 (1006–1171) µg/mL	20	<0.0001	Median (IQR)	Mann Whitney U test	at routine outpatient attendance	plasma	ELISA (Microvue SC5–9 Plus EIA)	
Ari et al., 2009 (46)	Factor H	933 ± 298 mg/L	21	1004 ± 342 mg/L	24	n.s.	Mean ± SD	one-way ANOVA	n.a.	serum	radial immunodiffusion, Binding Site (Human factor H NanoRID Kit, Birmingham, UK)	
Mei et al., 2017 (67)	Factor I	37.65 µg/mL	38	73.11 µg/mL	44	<0.05	Mean	t-test	n.a.	plasma	ELISA (CusAb Company, Wuhan, China)	
Blakey et al., 2023 (31)	Properdin	4828 ± 806 ng/mL	33	6877 ± 1421 ng/mL	33	<0.001	Mean ± SD	t-test	within seven days prior to delivery	plasma	electrochemiluminescent immunoassays (MesoScale Diagnostics (MSD, Rockville, MD))	
Blakey et al., 2023 (31)	Properdin	5282 ± 1467 ng/mL	35	7021 ± 1317 ng/mL	35	<0.001	Mean ± SD	t-test	within seven days prior to delivery	plasma	electrochemiluminescent immunoassays (MesoScale Diagnostics (MSD, Rockville, MD))	
Kolialexi et al., 2016 (68)	Vitronectin	44.04 (24.91–64.51) ng/mL	10	193.30 (118.92–508.73) ng/mL	40	<0.001	Median (IQR)	Mann Whitney U test	first trimester prenatal screening	plasma	ELISA (Mabtech, Sweden)	EOPE

EOPE, early-onset pre-eclampsia; IQR, interquartile range; LOPE, late-onset pre-eclampsia; n.a., not available; SD, standard deviation; SPE, severe pre-eclampsia. ^1^SPE defined according to the 2013 American College of Obstetricians and Gynecologists (ACOG) hypertension in pregnancy guidelines (40). ^3^SPE defined according to the 2019 ACOG hypertension in pregnancy guidelines (59).

The C markers can be divided into categories based on their involvement in the C cascade.

### 3.2 Classical pathway components

Fourteen studies (34.1%) were identified as measuring the blood concentrations of CP components, including C1q, C4, C4a, and C4d, as summarized in **Table 1**. Five studies measured C1q levels (12.1%), nine C4 (21.9%), one C4a (2.4%), and four C4d (9.7%). Regarding C1q, two studies reported no statistically significant differences in C1q levels between PE and control pregnancies (26, 28). One study showed significantly higher C1q levels in SPE (27). Two studies analyzing two different PE cohorts (EOPE and LOPE) reported significantly lower or higher C1q levels in PE, respectively (29, 30). Conversely, the majority of the studies were coherent with reporting a significantly lower level of C4 in PE women (29, 31, 32, 34, 35, 37), also when analyzing EOPE and LOPE separately (29). Only one article measured C4a levels, not detecting any significant difference in PE women, except for those experiencing the delivery of a small-for-gestational-age newborn (38). Four studies related to C4d fulfilled the inclusion criteria and suggested an increase in C4d levels in PE women (27, 30, 33, 39), particularly in LOPE (30).

### 3.3 Lectin pathway components

Our systematic review of currently available research highlighted very few studies (5/41; 12.1%) analyzing blood concentrations of LP components’ serum levels, as shown in **Table 2**. LP components were mainly measured in one study by Larsen and colleagues. They reported significantly higher serum concentrations of CL-L1, Map44, MASP-1, and MASP-3 in PE women than in healthy pregnancies, while significantly lower H- and M-ficolin levels. At the same time, no significant differences were observed in Map19, MASP-2, and MBL (41). MBL levels were further investigated in four additional studies (27, 28, 30, 42), but only one demonstrated an association between increased MBL levels and PE pregnancies (42).

### 3.4 Alternative pathway components

The vast majority of studies focused their interest on the assessment of AP components in serum or plasma samples (28/41; 68.3%), including Ba, Bb, C3, C3a, C3c, C3d, Factor B (FB), Factor D (FD), and iC3b, as reported in **Table 3**. Fragment Ba levels were measured by Blakey and colleagues in two different cohorts of PE patients matched with controls, reporting significantly higher levels of Ba in PE women only in one cohort (31). Seven studies (17.1%) related to Bb concentrations were evaluated, and they consistently demonstrated higher levels of Bb in PE, both in EOPE and LOPE (27, 29, 30, 33, 43–45). The central component C3 was analyzed in eight articles (19.5%), with a significant decrease in PE patients (29, 31, 33), even though not all studies reached statistical significance (32, 34–36, 46). Twelve articles (29.3%) measured C3a levels in the blood of PE women compared to healthy pregnancies, detecting an overall significant increase of C3a in PE (27, 30, 33, 39, 49, 50). Conversely, neither C3c (28, 53) nor C3d (36) plasma assessment showed significant differences between PE and normal pregnancies. FB was assessed by two studies, which determined a significant increase in serum levels (26) and not significant in plasma (28) of pregnant women with PE. Four studies consistently determined a greater concentration of adipsin/FD in serum (54, 55, 57) or plasma (56) of women with PE, particularly increased in SPE (54). Only one study by Blakey *et al.* reported a significant reduction of iC3b levels in PE (31).

### 3.5 Terminal pathway components

Terminal pathway components were investigated in 17/41 studies (41.5%) retrieved from the literature, mainly focusing on C5a and C5b-9 (**Table 4**). Circulating levels of the anaphylatoxin C5a were evaluated by twelve studies (29.3%). Most studies accordingly reported higher levels of C5a in the PE group (27, 30, 38, 48–51). Only one study by He and colleagues reported a significant reduction (28), while circulating C5a levels did not differ significantly between PE and control groups in the remaining studies (47, 52, 60, 61). Circulating levels of C5b-9 were measured by thirteen research articles (31.7%). Almost all of them were consistent with significantly higher concentrations of C5b-9 in blood samples of women with PE (27, 30, 33, 39, 49, 51, 54, 60–62). Interestingly, Burwick *et al.* analyzed C5b-9 in four different PE cohorts (PE, SPE, EOPE, and EOSPE), highlighting significantly higher plasmatic levels in PE compared to matched healthy groups, except for EOPE (62).

### 3.6 Complement regulators

Several studies (16/41; 39.0%) assessed the circulating levels of different C regulators, mainly focusing on C1-inhibitor and Factor H (FH), as summarized in **Table 5**. Four studies investigated the association between circulating C1-inhibitor levels and PE (33, 34, 63), but only one reported a significant reduction of C1-inhibitor in women with PE (37). Factor H levels were evaluated in seven studies, which determined an overall reduction of this C regulator in patients with PE (26, 28, 29, 46, 47, 66) in both EOPE and LOPE (29). A statistically significant reduction in PE cohorts was observed also for Factor I (FI) (67), properdin (31), and vitronectin (68). Velasquez and colleagues reported higher plasma levels of CD59 in PE (64). Conversely, C4b-binding protein (C4BP) (33) and complement receptor 1 (CR1)/CD35 (65) did not show any statistical differences in PE and control groups.

## 4 Discussion

### 4.1 Overview of the complement components in pre-eclampsia

Even though C dysregulation may have a role in affecting placental formation before the disease onset, the contribution of C in PE pathophysiology mainly consists in a secondary mechanism of amplification of tissue injury and inflammation, following endothelial damage and local placental ischemia and hypoxia, and results in a cascade of reactions that contribute to the rapid development and symptom exacerbation of PE at the systemic level (69, 70). When discussing C testing, one should always bear in mind the complexity of C and the interdependence among its individual components. For ease of comprehension, our systematic review dissected the involvement of C components as biomarkers of PE, initially focusing on the contribution of the different C pathways as separate entities, and then moving to a more extensive overview.

As regards the CP and LP, studies were coherent in reporting lower levels of C4 and higher levels of C4d in PE compared to healthy pregnancies. This supports the hypothesis that the relatively low C4 values in PE may indicate a low grade of CP and LP activation with consumption of C4, as suggested by the increase in C4d levels. Agostinis and co-authors also demonstrated that lower levels of C4 were associated with PE (71). Consistently, excessive deposition of C4d at the placental site was frequently reported in PE (23, 31, 72) as a marker of CP and LP activation, even though C4d deposits seem more likely related to CP activation due to the co-localization with C1q (73). The vast majority of the resulting proteolytic cleavage product C4d remains at the activation site, while part of it may eventually enter the circulation as free C4d.

The CP or LP may be triggered by repeated ischemia–reperfusion injury and oxidative stress caused by defective placentation in PE (72, 73), while AP exacerbates the activation cycle via an amplification loop. In human PE, C has been shown to be locally activated via the LP triggered by H-ficolin, while MBL did not appear to be particularly involved (23). Larsen and colleagues reported that LP protein concentrations in the serum generally increased throughout the trimesters of normal pregnancy and reduced after delivery in healthy pregnant women and in women with PE (41). A study by Celik and Ozan also reported higher MBL levels in SPE patients compared to women with uncomplicated pregnancies (74), in accordance with further studies (42, 75). Conversely, herein reported results about C1q levels were inconclusive. Dijkstra and colleagues observed no significant differences in serum C1q between PE and control pregnancies in three different cohorts (76),while Agostinis *et al.* reported a decrease in PE patients, both in serum and plasma samples, maybe due to C1q binding to circulating immunocomplexes or to syncytiotrophoblast extracellular vesicles derived from PE placentas (71, 77, 78). C1q reduction is also supported by the findings of Jia *et al.*, both in EOPE and LOPE (29). In the occurrence of PE related to antibody-mediated autoimmune diseases (*e.g.*, antiphospholipid syndrome), C1q reduction could be associated with CP activation induced by the deposition of pathogenic immunoglobulins in the placenta (79).

The AP is also consistently activated in PE, as demonstrated by the reduction in circulating C3 levels and the increased levels of Ba, Bb, and C3a fragments, being markers of AP activation. Similar results are also consistent with the higher levels of circulating FD in PE (54–57), which is responsible for excessive C activation. Interestingly, the increase in Bb levels is consistent with increased FD levels in PE, considering that FD serves as a serine protease acting on FB to yield the active fragment Bb (57). It is also noteworthy to consider that FD levels may increase in parallel to decreased kidney function and be responsible for enhanced AP activation, particularly in the circulation (80). Elevated circulating levels of C3a in the first trimester of pregnancy were demonstrated as an independent predictive factor for PE (45). Moreover, C3a levels were particularly increased in LOPE (30). Accordingly, local C3 deposition in the placentae of PE patients is significantly higher compared to the control placentae (81).

Evidence about terminal pathway components in PE is rather coherent among studies, reporting increased C5a and C5b-9 levels in the maternal circulation, both in EOPE and LOPE (30, 49). Interestingly, a significant correlation was found between C5b-9 levels and anti-angiogenetic factors (*i.e.*, soluble endoglin and sFlt-1). We should keep in mind that the increase in C5a and C5b-9 may also be directly influenced by additional factors, such as coagulation and fibrinolysis. Thus, urinary excretion of C5b-9 has been proposed as a more valuable marker of SPE than plasma evaluation (51).

Taken together, existing data show that C regulation is also impaired in PE. During normal pregnancy, FH levels usually increase throughout trimesters of healthy pregnancies (28), suggesting that a balanced regulatory response is crucial to avoid excessive systemic AP activation, despite allowing local C3-dependent mechanisms required to support a normal pregnancy. Interestingly, three studies consistently reported that circulating levels of FH were significantly reduced in PE (29, 47, 66). Yasmin et al. identified lower FH levels in sera collected in the first trimester of pregnancies with PE, proposing FH as a potential predictive biomarker of PE (66). The relative abundance of FH in the third trimester of pregnancy, and particularly the increased FH/C3 ratio, could prevent AP activation, while a reduction in FH may participate in the C dysregulation observed in PE. This scenario is also consistent with an overall reduction of other C regulators, *i.e*., properdin (31) and FI (67). Reduced concentrations of properdin in maternal plasma allowed to distinguish cases of PE from healthy pregnancies with excellent diagnostic accuracy (31). Excessive AP activity may determine reduced circulating levels of properdin via consumption from tissue deposition.

The overall emerging scenario is an abnormal C activation and regulation in PE. Decreased circulating levels of C1q are frequently observed in combination with lower levels of C4, which may be due to increased consumption of C4 via aberrant activation of the CP or LP. Reduced concentrations of early AP components (*i.e.*, C3, FB, or FH) are also consistent with an extensive depletion of the cascade components due to excessive activation or poor inhibition, while C activation products (*e.g.*, Bb, C3a, C5a, or C5b-9) are increased as a result of the extensive cleavage of C components. A pivotal role in the dysregulation of C activation seems to be carried out by the reduction in FH levels, which could be caused by deficiency or consumption of FH. Interestingly, a recent study by Lokki et al. identified FH variants that predispose to PE onset, confirming the multifactorial nature of the pathophysiological mechanisms underlying PE (82).

Jia and colleagues demonstrated that the best predictive indicator of PE was the combination of five C factors (C1q, Bb, FH, C3, and C4), both in EOPE and LOPE, displaying a potential as diagnostic markers for severe PE (29).

### 4.2 Pitfalls of complement testing in pre-eclampsia

An accurate analysis of a wide range of C components in PE is of utmost importance for prediction and diagnosis, although the reliability of these measurements is often challenging. The main concern of C assessment for diagnosing disease and monitoring therapy is that measured concentrations of C components may widely vary among different laboratories due to a lack of protocol uniformity for pre-analytical sample handling (*e.g.*, collection, processing, and storage), use of different calibrators and techniques (*e.g.*, nephelometry, turbidimetry, and ELISA), or antibodies targeting different epitopes. These pitfalls unveil an urgent need for standardization, in concert with adequate pre-analytical sample handling and storage. However, up to now, performing ELISA with well-defined antibodies has proven to be the most reproducible method for reflecting the actual state of C activation *via* quantification of C-derived split products next to the native proteins, while nephelometry and turbidimetry do not allow to distinguish between the native non-activated C proteins and their activated split products (83).

Furthermore, laboratories do not always analyze the proper sample type for C analysis. The use of plasma or serum can lead to different results when certain C factors are considered. In particular, plasma is preferentially chosen for assessing C byproducts to avoid *in vitro* C activation that occurs during serum preparation due to coagulation and fibrinolysis activation. EDTA-plasma (at least a final concentration of 10 mM), any activation of C is minimized (83). For other factors, the binding of C components to fibrinogen and fibrin in plasma should be taken into account (*e.g.*, C1q) (84). Inconsistences may also be due to sample storage, thawing conditions, and repeated freezing-thawing cycles, which could be crucial variables for specific C components, particularly activation products (85).

Non-uniformity in the timing of sample collection is another issue that needs to be discussed when examining PE, and a serious limitation when comparing the studies collected in this systematic review. Although it may be assumed that the closer the disease onset, the more the potential biomarkers are expected to vary, a different matter is the potential predictive value of the C-components in PE. It is noteworthy that the vast majority of studies gathered in the current review measured C-component expression at the time of PE diagnosis (*i.e.*, in the third trimester of pregnancy). Only a few studies also measured C-components during the first trimester, with limited but encouraging results. He et al. observed significant fluctuations in circulating levels of FB, FH, C1q, C3c, and C4 in PE pregnancies from the first trimester onwards (28). Altered FD and C5a levels in women with PE in the first trimester were also reported (56), as well as FH (66). These preliminary findings emphasize the urgent need to investigate variations in the C components during pregnancy to identify potential predictive markers at early stages. As PE can have multiple causes, it is unlikely that a single timing strategy for biomarker testing can be used to predict all cases of PE (86). Despite requiring a massive effort from clinicians and researchers in terms of number of enrolled women, it is crucial that we embark on prospective longitudinal studies involving blood sample collection at various stages of pregnancy. These studies may allow us to explore the actual time threshold of biomarkers with predictive significance and ensure proper matching for gestational age between healthy and pregnancies with PE.

Diverging results among studies may also be biased by the cohort selection due to different ethnicity, sample size, symptom severity (mild/severe), onset (preterm/term), and presence of comorbidities. For instance, women with African ancestry are at greater risk of PE, likely due to the involvement of specific factors, among which Bb fragment has been accounted (43), while factor B activation has not been observed in specific Caucasian cohorts (33). This evidence suggests that some C factors may serve as predictors of PE only in specific sub-groups of patients, offering a promising avenue for further investigation and potentially explaining the variations among the cohorts analyzed in this review.

Several lines of evidence support the concept that PE should not be considered a single disorder, and the guidelines no longer support the classification into “mild” and “severe” PE. Therefore, the definition of PE severity may not be consistent across studies, based on different updates of the American College of Obstetricians and Gynecologists (ACOG) hypertension in pregnancy guidelines (40, 58, 59), and may lead to bias in the cohort selection. It is also worth considering that EOPE and LOPE may be explored as separate entities reflecting underlying differences in etiology. The complexity of defining and classifying PE was further highlighted by Than *et al.*, who described four molecular clusters of PE (i.e., canonical/placental, metabolic, immunological, and maternal PE) by proteomic analysis that exhibit distinct clinical phenotypes (87), in line with maternal hemodynamic characteristics of different hypertensive disorders of pregnancy phenotypes (88).

The presence of comorbidities is a crucial factor that must be taken into consideration. Interestingly, several studies that were specifically excluded from the current systematic search report the usefulness of C-component assessment in specific subgroups of patients. For example, predictive value was found for FB and FH serum levels in PE associated with gestational diabetes mellitus (89).

### 4.3 Strengths and limitations of the systematic literature review

To our knowledge, this is the first systematic literature review focusing on currently available research about the assessment of C components as circulating biomarkers of PE. The main strength of the current systematic search includes the comprehensive coverage of available measurements of circulating C components in PE, which were summarized as ready-to-use information.

Nevertheless, we can recognize some methodological limitations. First, not all scientific information may be included in bibliographic databases being reported as unpublished conference papers or congress proceedings. In addition, we included only research papers in which numerical values for the measurement of the C-component were reported, thus possibly neglecting other seminal studies in which numerical values were not explicitly reported. Lastly, only publications in English were included, which may lead to language and country-specific biases, potentially limiting the generalizability of our findings.

## 5 Conclusion and perspectives

Since C activation can occur in several scenarios, the specificity of a single C-component test is low, so a panel of tests should be preferred (90). C multiplex ELISA assays, such as panels for simultaneous testing of C activation fragments and intact components or regulators (91), may be particularly advantageous from a technical and reproducibility standpoint. Thus, multiplex assays can help to address the need for a thorough analysis of the status of C activation and regulation and ensure a comprehensive overview of the C cascade. Here, we propose that multiplex C testing should be performed throughout pregnancy to raise awareness of the importance of planning prospective longitudinal studies to identify early predictive markers for PE.

With the mounting evidence of C activation, the potential of C-targeted therapy for the treatment of PE is a promising avenue. The emergence of new drugs specifically targeting C underscores the growing necessity for soluble biomarkers as companion tests to select and monitor patients who would benefit from these innovative treatments, instilling hope for improved outcomes in the management of PE.

## Data availability statement

The original contributions presented in the study are included in the article/**Supplementary Material**. Further inquiries can be directed to the corresponding author.

## Author contributions

AB: Conceptualization, Data curation, Funding acquisition, Methodology, Writing – original draft, Writing – review & editing. CA: Conceptualization, Data curation, Funding acquisition, Writing – original draft. AM: Data curation, Methodology, Writing – original draft. GZ: Writing – review & editing. TS: Writing – review & editing. GR: Funding acquisition, Supervision, Writing – review & editing. RB: Conceptualization, Supervision, Writing – review & editing.
