# Supplementary material for: Systematic review of the complement components as potential biomarkers of pre-eclampsia: pitfalls and opportunities
Source: Front Immunol. 2024 Jun 24;15:1419540. doi: 10.3389/fimmu.2024.1419540 (PMC11232388; doi:10.3389/fimmu.2024.1419540)
Supplement: Supplementary file 1 [file Table_1.docx]

**Supplementary Material to**

**Systematic review of the complement components as potential biomarkers of pre-eclampsia: pitfalls and opportunities**

**Andrea Balduit^1^, Chiara Agostinis^1,*^, Alessandro Mangogna^1^, Gabriella Zito^1^, Tamara Stampalija^1,2^, Giuseppe Ricci^1,2^, Roberta Bulla^3^**

^1^ Institute for Maternal and Child Health - IRCCS “Burlo Garofolo”, Trieste, Italy

^2^ Department of Medical, Surgical and Health Science, University of Trieste, Trieste, Italy

^3^ Department of Life Sciences, University of Trieste, Trieste, Italy

*** Correspondence:** Chiara Agostinis ([cagostinis@units.it](mailto:cagostinis@units.it)). Institute for Maternal and Child Health - IRCCS “Burlo Garofolo”, Trieste, Italy. Phone: +39 040 5588652.

Keywords: pre-eclampsia; complement system; systematic review; biomarker, pregnancy.

**Running title:** Complement components in PE.

**Search strategy**

| **Databases** | PubMed/Medline, Scopus, Embase | |
| --- | --- | --- |
| **Total** | **812** | |
| **Database** | **n** | **Search** |
| **PubMed** | 205 | ((complement[Title/Abstract]) AND (preeclampsia[Title/Abstract])) AND ((serum[Title/Abstract]) OR (sera[Title/Abstract]) OR (plasma[Title/Abstract]) OR (blood[Title/Abstract]) OR (biomarker[Title/Abstract])) |
| **Scopus** | 176 | ((TITLE-ABS(complement) AND TITLE-ABS(preeclampsia) AND TITLE-ABS(biomarker))) OR ((TITLE-ABS(complement) AND TITLE-ABS(preeclampsia) AND TITLE-ABS(plasma))) OR ((TITLE-ABS(complement) AND TITLE-ABS(preeclampsia) AND TITLE-ABS(blood))) OR ((TITLE-ABS(complement) AND TITLE-ABS(preeclampsia) AND TITLE-ABS(serum))) AND TITLE-ABS(preeclampsia) AND TITLE-ABS(sera))) |
| **Embase** | 431 | complement:ab,ti AND preeclampsia:ab,ti AND (serum:ab,ti OR sera:ab,ti OR biomarker:ab,ti OR blood:ab,ti OR plasma:ab,ti) |
